# An Extended Twin-Pedigree Study of Neuroticism in the Netherlands Twin Register

**DOI:** 10.1007/s10519-017-9872-0

**Published:** 2017-10-17

**Authors:** Dorret I. Boomsma, Quinta Helmer, Harold A. Nieuwboer, Jouke Jan Hottenga, Marleen H. de Moor, Stéphanie M. van den Berg, Gareth E. Davies, Jacqueline M. Vink, Maarten J. Schouten, Conor V. Dolan, Gonneke Willemsen, Meike Bartels, Toos C. E. M. van Beijsterveldt, Lannie Ligthart, Eco J. de Geus

**Affiliations:** 1 Netherlands Twin Register, Department of Biological Psychology, Vrije Universiteit Amsterdam, Van der Boechorststraat 1, 1081 BT Amsterdam, The Netherlands; 2 EMGO+ Institute for Health and Care Research, Vrije Universiteit Amsterdam, Amsterdam, The Netherlands; 3 Avera Institute for Human Genetics, Sioux Falls, USA; 4 Clinical Child and Family Studies, Vrije Universiteit Amsterdam, Amsterdam, The Netherlands; 5 Department of Research Methodology, Measurement, and Data Analysis, University of Twente, Enschede, The Netherlands; 6 Behavioural Science Institute, Radboud University, Nijmegen, The Netherlands

**Keywords:** Pedigree analysis, Neuroticism, Heritability, Genetic non-additivity, Shared household effect, Extended twin-family design, Netherlands Twin Register

## Abstract

For the participants in the Netherlands Twin Register (NTR) we constructed the extended pedigrees which specify all relations among nuclear and larger twin families in the register. A total of 253,015 subjects from 58,645 families were linked to each other, to the degree that we had information on the relations among participants. We describe the algorithm that was applied to construct the pedigrees. For > 30,000 adolescent and adult NTR participants data were available on harmonized neuroticism scores. We analyzed these data in the Mendel software package (Lange et al., Bioinformatics 29(12):1568–1570, 2013) to estimate the contributions of additive and non-additive genetic factors. In contrast to much of the earlier work based on twin data rather than on extended pedigrees, we could also estimate the contribution of shared household effects in the presence of non-additive genetic factors. The estimated broad-sense heritability of neuroticism was 47%, with almost equal contributions of additive and non-additive (dominance) genetic factors. A shared household effect explained 13% and unique environmental factors explained the remaining 40% of the variance in neuroticism.

## Introduction

Like many large twin registries worldwide, The Netherlands Twin Register (NTR) not only recruits twins, but also their family members, including parents, siblings, spouses and offspring of twins. This has led to a database with roughly equal proportions of participants who are and who are not twins. In genetic studies, the simultaneous analysis of all data from extended twin families can offer substantial gains in statistical power, when, for example, estimating non-additive genetic components of variance or when considering the effects of a shared family environment (Posthuma and Boomsma 2000; Rebollo and Boomsma 2006a, b; Keller et al. 2010). Here, we analyzed a large dataset on neuroticism from twins, their biological relatives and non-biological family members, including spouses (N > 30,000 subjects), after constructing the extended pedigrees (see “Methods”), that link all nuclear families from the Netherlands Twin Register to each other within larger pedigrees. Neuroticism questionnaires were completed by twins and their spouses, their parents and siblings in a number of NTR projects (e.g. Middeldorp et al. 2007; Nivard et al. 2015). Two different personality instruments were used and data for neuroticism were harmonized using an item-response theory (IRT) approach (van den Berg et al. 2007, 2014). Van den Berg et al. (2014) applied IRT to neuroticism data from a large number of international cohorts with twin data (total N = 29,496 twin pairs from six cohorts) and estimated the heritability of neuroticism at 48% with roughly equal contributions of additive and non-additive genetic factors. This estimate closely resembled the estimate of 42% from a large meta-analysis of behavior genetic studies on personality including over 100,000 subjects (Vukasović and Bratko 2015). Moderator analyses in this meta-analysis indicated higher heritability estimates in twin studies (47%) compared to family and adoption studies (22%). Vukasović and Bratko (2015) attributed the difference in these estimates to non-additive genetic effects, which contribute to resemblances of monozygotic twins and full siblings, but not to resemblance of other family members, with the exception of double first cousins (Weir et al. 2006). The contribution of shared environment to familial resemblance was not considered in the meta-analysis. Given data obtained in nuclear families (including twins), it is difficult to assess the importance of shared environment (Keller et al. 2010). Here we modeled neuroticism data from twins and their relatives in extensive pedigrees, which allowed estimation of both non-additive genetic and shared environmental effects.

In genetic covariance modeling, the specification of relationships among family members and the calculation of pedigree likelihoods given large and irregular pedigrees is not straightforward. Twin data are often analyzed in structural equation modeling (SEM) software, such as Mx, OpenMx, Mplus, or LISREL (Neale et al. 1994, 2016; Muthén et al. 2006; Jöreskog 1970). While these programs offer great flexibility in model specification, they are less well suited to the analysis of data from large and complex pedigrees. As an alternative, we used the Mendel software package (Lange et al. 2013) for genetic data analyses. We constructed and implemented an algorithm which took the NTR administrative database (Boomsma et al. 2008) as input and gave the larger pedigree structures in standard pedigree format as output to be used by software such as ‘Mendel’ (Lange et al. 2013) or ‘Merlin’ (Abecasis et al. 2002). The Mendel software constructs the genetic relations among pedigree members, e.g. sibling, cousins, aunt–niece, grandparent–grandchild etc., and estimates components of genetic covariance for any pair of relatives from the weighted combination of additive and dominance effects (Weiss 1993). The information required to reconstruct genetic relationships between individuals in a pedigree of any size is (1) a family identifier (ID) shared by all members of a pedigree, (2) an individual identifier, (3) a link to the parents of that individual, (4) and the individual’s sex, appropriately coded. Genetic relations among members of a pedigree who share the same family ID are traced by specifying the parents of individuals. A pedigree is defined by a set of related individuals, in which each individual either is either a founder (no parents in the pedigree) or not. Both parents of non-founding individual are identified within the pedigree. Additionally, extra fields can be added to indicate the presence of monozygotic twins (or higher-order multiples) in a pedigree and to indicate whether persons share the same household within a pedigree, regardless of the genetic relations among the individuals who share a household. However, whereas genetic relations do not change, household sharing can depend on the time of assessment of the phenotype. Notably in longitudinal studies, offspring may share a household with their parents or with their parents and siblings at the start of the study, but establish their own households later on. Here we first present an algorithm, which is available from the first author, that we developed to generate a pedigree file for all participants in the Netherlands Twin Register. Next, we used the Mendel software package (https://www.genetics.ucla.edu/software/Mendel_current_doc.pdf) for the genetic analysis of neuroticism, specifying models that included variance components due to additive and non-additive genetic factors as well as to shared household.

## Methods

### Participants

Participants in the Netherlands Twin Register are young and adult twins, their biological and non-biological family members and their teachers. The Person Administration of the Netherlands Twin Register (PANTER) database is a person-oriented database that stores and specifies any number of relationships between persons (Boomsma et al. 2008). The PANTER database currently stores information on 275,250 participants. Between participants, there can be parent–offspring relationships, sibling–sibling, spouse–spouse, twin–twin and teacher–student relations. Each individual can have an unlimited number of one-to-one relations with other individuals in the database. For example, multiple spouse–spouse links are possible, as are multiple father–offspring relations. A woman can have mother–offspring relations as well as teacher–pupil relations. The PANTER database stores all relations among participants, with information on whether relations are either social or biological stored in different databases. Examples of social, non-biological, relations include person A being registered as a child of person B, while person A was adopted; (nearly) all spouse–spouse relations (marriages between cousins are allowed in the Netherlands); or person A being the offspring in a family where the mother married for a second time and person B is the second husband of A’s mother, so that A and B have a parent–offspring link. In PANTER, both the first and second husbands of mother can have a parent–offspring relation with person A. Additional NTR databases contain information on pedigree relations from demographic databases, genotyping projects, surveys and interviews and specify information regarding the zygosity of twins and biological or social relations among family members. Zygosity information comes from blood group or DNA markers or survey data and consists of one-to-one relations describing whether co-twins are monozygotic (MZ) or dizygotic (DZ). For our study of neuroticism, all relationship types except teacher–student relations are relevant.

### Measures of neuroticism

Neuroticism was assessed by the Amsterdamse Biografische Vragenlijst (ABV, Wilde 1970), and the NEO Five-Factor Inventory (NEO-FFI, Costa and McCrae 1992). The ABV was part of adolescent and adult NTR surveys collected in 1991, 93, 97, 2000 and 2002 and is a Dutch personality questionnaire that was closely modeled on the Eysenck Personality Questionnaire (EPQ). The NEO-FFI was included in adult NTR surveys collected in 2004 and 2009 and in adolescent NTR surveys (Bartels et al. 2013). Neuroticism data were harmonized to a common scale by IRT modeling (van den Berg et al. 2014). For each subject one IRT score was available and the age at which this score was measured. There were 31,152 NTR participants (41% male) with valid neuroticism data. Their average age when Neuroticism was assessed was 37.26 (SD = 15.34) years (37.8 in men, 36.9 in women) and year of birth ranged between 1909 and 1997 (mean 1965). Twins and multiples made up 47% of the total sample. Most twins came in complete pairs (85% of the sample), 14.4% came from incomplete pairs and the remaining were higher order multiples.

### Genetic analyses

To obtain a first impression of familial resemblance for Neuroticism, correlations were estimated for MZ and DZ twin pairs, for biological sibling pairs, for father–offspring and for mother–offspring pairs. Two spouse correlations were computed: one for parents of twins and one for twins and their spouses, who on average were 17 years younger than the parents of twins. Familial correlations were obtained for independent pairs of relatives, e.g. a family with three siblings provided one sibling pair. Father–offspring and mother–offspring pairs similarly were based on selecting different twins from a pair. The 95% confidence intervals (CI) around correlations were obtained from Mx (Neale and Miller 1997). Genetic analyses, aiming at estimating total and narrow-sense heritability and the effects of sharing a household, were carried out in Mendel (Lange et al. 2013). In all analyses, age and sex were included as fixed effects. The variance of the residuals of the linear regression model was decomposed into genetic and environmental components. We estimated additive (A) and non-additive or dominance (D) genetic variance and also included variance due to shared household effects. Sharing a household was defined for all spouses living together and for parents with their offspring when the offspring was 18 years old or less. The remainder of the variance was environmental variance not shared by family members (E). Phenotypes of individuals who were not part of the same extended pedigree were assumed to be uncorrelated. We analyzed three models with different combinations of the A, D, household and E variance components and tested the significance of variance components due to household and non-additive genetic factors. We note that the likelihood-ratio test of a variance component does not follow the usual central χ^2^(1) distribution under the hypothesis that the variance component is zero (Dominicus et al. 2006) and adopt the following simple procedure: we test the variance component using the standard test, recognizing that this results in an inflated type I error rate. This is problematic only if one does not reject the alternative hypothesis that the variance component is zero.

**Fig. 1:**
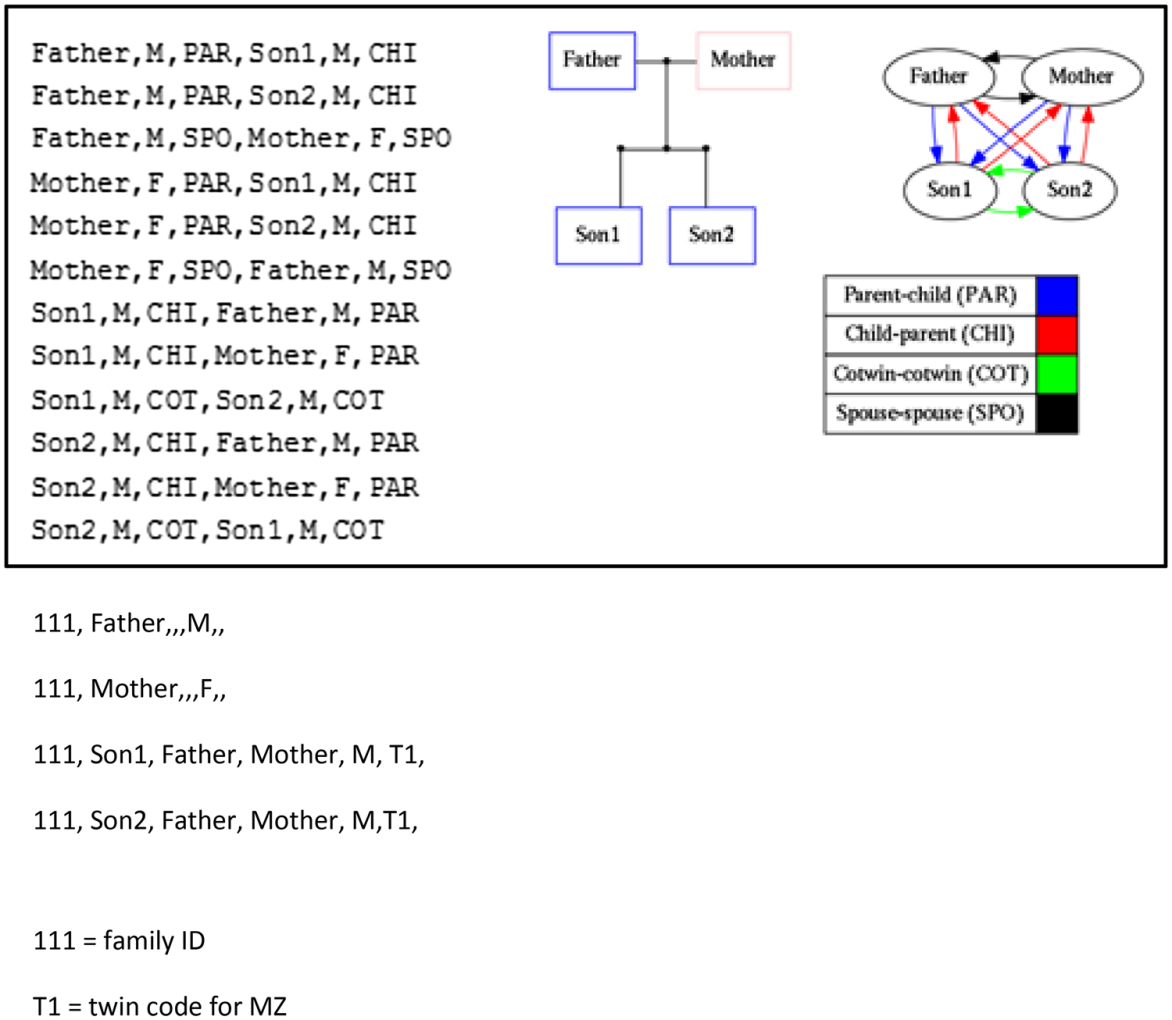
Specification of relations among NTR participants in the PANTER database for a nuclear family of two parents with MZ twin boys, below the specification of this pedigree in the MENDEL input file

We defined a nuclear family as consisting of a male and female parent, and all known offspring who share these parents. The offspring can have their own spouses and their own offspring with their spouse. A family consists of all individuals who are known to be related in some way, and will generally consist of one nuclear family or the union of multiple nuclear families, linked through parent–child relationships, sibling–sibling relations or spouse–spouse relations. Individuals can have multiple spouses, with whom they do or do not have offspring. The relational data required for the pedigree construction were extracted from the PANTER database, in the form of a file listing all existing one-to-one relationships between individuals in the database, as well as their gender and the types of relationship (e.g. spouse–spouse, parent–offspring). The data for pedigree construction are retrieved every 24 h from the PANTER database. These data are then combined with other data sources, such as the zygosity database. Information on relations that are known to be non-biological is also imported from external databases and used in the pedigree buildup. Mendel’s input pedigree file consists of 6 required fields per individual, plus additional fields for shared household effects, for quantitative traits and discrete variables. The six basic fields describe the family ID, the ID of the individual, the ID of the father of that person, the ID of the mother, the gender of the individual and a monozygotic twin (or higher-order MZ multiple) identifier. The MZ identifier is an ID indicating that every person with this specific identifier in the family is monozygotic with another person, or persons, sharing that identifier. All family members are grouped together in the input (Fig. 1). Household identifiers can be added as extra columns indicating that people with the same label in this field share a household, which may increase their phenotypic resemblance. A household indicator may specify spouse pairs, each member of a pair sharing an identifier with the spouse, with or without their offspring sharing a household, or for example specify that all twins, regardless of zygosity, or all offspring regardless of twin or non-twin status share an environmental component. Individuals are required to have either both or neither of their parents identified. To deal with missing parent–offspring relations, the creation of “dummy” parents was necessary, i.e. parents who define a nuclear family but who are not registered with the NTR. Also, the presence of more than two registered parents must be handled. More than two parents in a family occur when parents of twins divorce and then remarry. NTR has several instances where the new marriage then also has twin offspring. The algorithm for dummy-parent creation is as follows: Consider an individual called P. When P is a founder (i.e. an individual without registered parents) and P has no siblings or all of P’s siblings are founders as well, create two dummy parents for P and P’s siblings and co-twins. In case P is a founder but any of P’s siblings is not, consider two different situations. In the first situation, P has a registered co-twin and is assigned the same parents as P’s co-twin. In the second situation, P only has non-twin siblings and P is to be removed from the pedigree. Move on to the next individual and repeat this until all individuals have been processed. If P has more than two registered parents, e.g. two fathers and a mother, and it is not known which father is the biological parent, remove P from the pedigree and move on to the next individual. If a co-twin of P has parents who do not match with the parents of P, remove P and move on (here we assume that two twins always share the same parents, this is not assumed for siblings). No parents need to be created for P if exactly two parents of opposite sex are registered for P. Finally, one dummy parent needs to be created for P and all of P’s co-twins and biological siblings in case only one parent is registered. The approach implies that for families where we cannot establish genetic relations, e.g. from survey or other additional data, that these families are removed. When the family structure was unclear, additional data regarding complex parent–child and sibling–sibling relations were obtained from the Dutch city council administration (Gemeentelijke Basisadministratie: GBA), information was checked against the NTR genotype database and information from surveys was utilized, where NTR participants were asked about biological and social relations with other family members. We removed 135 subjects from the pedigrees for whom biological versus social relationship could not be resolved. In the pedigree construction we also removed all teachers of twins (i.e. teacher–student relations are not included).

## Results

The NTR pedigree consisted of 253,015 individuals in 58,645 extended families (excluding 7201 “dummy” parents created to specify the pedigree structure). There were 14,805 MZ twin, or triplet, multiples, 6626 were male and 8179 female. The number of nuclear families in the NTR pedigree was 59,674, with the largest nuclear family consisting of 14 individuals. A nuclear family contained at most seven daughters or at most 11 sons. On average, a nuclear family contained 2.29 children, for a total of 136,737 offspring. Of these children, 69,301 were female, and 65,119 were male (for 2,307 twins and ten others sex was unknown). For the genetic analyses of neuroticism in Mendel a total of 36,639 individuals were present in a trimmed pedigree of which 31,152 were phenotyped (a large number of twin families in NTR come from the Young NTR in which phenotyping for personality is limited). The number of nuclear families with neuroticism data and with at least two family members was 7854. Table 1 describes the composition for the entire NTR pedigree and for the subset with neuroticism data. The Mendel software allowed us to analyze more the complex family structures, providing extra sources of information for heritability-related analyses beyond parents and twins. An example of a more complex family structure in the NTR pedigree is a ‘double first cousin’ structure. Here, two dizygotic twin pairs A1, A2 and B1, B2 were registered as spouses (i.e., A1 and B1 were registered as spouses, and A2 and B2 were registered as spouses). Both couples had offspring (one and two children respectively), so that these offspring had the same set of grandparents, even though they had different parents. There was information on 87 aunts and uncles of twins, and a large (34 members) extended pedigree where cousins of twins were also measured. This is not the largest pedigree in the set, which is one with 44 members. In total there are 514 large (> 10 family members) and 40 very large (> 20 family) pedigrees.

**Table 1:** Number of registered individuals in the Netherlands (excluding teachers^a^)

	NTR pedigrees	Neuroticism pedigrees
Number of extended families	58,645	9527
Individuals	253,015 registered	36,639 (31,152 phenotyped)
MZ male (individuals)	13,268	2451
MZ female (individuals)	16,387	4904
MZ (individuals^b^)	**29,657**	7355
DZ male (individuals)	33,860	3163
DZ female (individuals)	33,659	4625
DZ (individuals)	**67,519**	7788
Unknown zygosity male (individuals)	10,402	192
Unknown zygosity female (individuals)	10,588	390
Unknown zygosity (individuals^c^)	23,297	582
Fathers	57,010	5678
Mothers	57,872	5992
Parents^d^	**114,882**	11,670
Brothers	7214	1586
Sisters	8369	2811
Full sibs (non-twin individuals^e^)	**15,589**	4397
Half-brothers	167	29
Half-sisters	176	54
Half-sibs (non-twin individuals^f^)	**343**	83
Spouse pairs with offspring^g^	56,795 pairs	5550 pairs
Spouse pairs without offspring^h^	3089 pairs	2556 pairs

Bold numbers represent the total number of MZ and DZ twins, parents, sibs and half-sibs

^a^NTR also includes 21,478 teachers who provide data on twins; 622 double registrations and 135 individuals for whom the biological parents cannot be resolved. These were removed from the pedigree

^b^Includes 2 MZ transgender individuals

^c^Includes 2307 individuals of unknown gender

^d^Parents: individuals with at least one offspring (7201 dummy parents are not counted)

^e^Six siblings of unknown sex

^f^Half sibs: all individuals who (pairwise) share one biological parent; e.g. families with twins and one-half-sib are counted as one; families twins, one-half-sib en two full sibs are counted as three

^g^Spouse pairs with offspring: two persons with at least one offspring (dummy parents excluded)

^h^Spouse pairs without offspring: spouses without registered offspring or parents

Table 2 summarizes correlations for neuroticism among family members. Correlations were highest for MZ twins (0.52), very similar for DZ and sibling pairs (0.22 and 0.20) and lower for parents and offspring (0.14 for father–offspring and 0.16 for mother–offspring). The spouse correlations in parents of twins, who were on average 52 years old, and in twins and their spouses, who on average were 35 years old, were 0.16 and 0.05, respectively, suggesting that sharing a household for more prolonged periods of time increases resemblance for neuroticism. The pattern of familial correlations is consistent with at least three, not mutually exclusive, hypotheses: a lower parent–offspring than sibling correlation is expected when genetic dominance contributes to phenotype variation (Lynch and Walsh (1998). Second, this pattern of resemblance is also expected under genotype by age interaction and, thirdly if there would be household by age interaction. However, only the first hypothesis is consistent with a difference between MZ and DZ correlations that is larger than twice the correlation in DZ pairs, which is the pattern observed in the data.

**Table 2:** Basic summary of familial correlations and their confidence intervals for neuroticism

	N Complete pairs	Correlation	95 CI	Average age
MZ twins	2984	.523	.499–.545	30.1
DZ twins	3121	.216	.182–.248	27.2
Siblings	596	.198	.120–.271	38.3
Father-twin1	3580	.143	.111–.174	53.5/23.6 (father/twin)
Mother-twin2	4232	.163	.133–.191	51.6/23.6 (mother/twin)
Parents of twins	3847	.162	.131–.192	53.3/51.4 (father/mother)
Twin-spouse	1845	.053	.007–.098	34.6/35.6 (twin/spouse)

The hypothesis of genotype by age interaction would be consistent with parents and offspring who differ more in age being less similar in their neuroticism scores than parents and offspring who are closer in age. We computed the differences in age between parents and offspring at the age at which they completed a neuroticism survey and the difference in their neuroticism scores. Distributions of these difference scores are given in the appendix. The average age difference for the father–offspring pairs was 29.8 years and for mother–offspring 28.1 years, with a substantial spread in age-difference scores in both groups. Parent–offspring pairs who did not differ by age when they completed the survey reflect those pairs in which father completed a neuroticism survey in e.g. 1991 and his son or daughter in 2013, both at the age of 22 years. The oldest age at birth of offspring was 53 for fathers and 43 years for mothers. There was no correlation between age-difference and neuroticism-difference scores in father–offspring pairs (r = − 0.014, p = .39), and a small negative correlation in mother–offspring pairs (r = − 0.068, p < .00), i.e. larger age differences were associated with smaller differences in neuroticism, which seems not compatible with a genotype age interaction effect. The analysis was repeated in 514 sibling pairs, whose average age difference when the completing the neuroticism questionnaire was 4.02 years (SD = 3.30), with the largest age difference being 27 years. No correlation was seen in the sibling group between age-difference and neuroticism-difference scores (r = 0.007, p = .87). The shared household by age interaction hypothesis was explored by looking at parent–offspring correlations in families with offspring ages 18 years or younger versus parent–offspring correlations in families with offspring over 18 years. For father with offspring the correlation was 0.20 (0.13–0.27) in 605 families with offspring 18 years or younger, and 0.13 (0.09–0.16) in 2975 families with offspring over 18 years. For mother–offspring, the correlation was 0.19 (0.12–0.25) in 768 families with offspring 18 years or below, and 0.16 (0.13–0.19) for offspring over 18 years from 3464 families. This pattern is consistent with younger children, sharing a household with their parents, resembling their parents more, but CI in the younger group are relatively large. For MZ and DZ twins a similar breakdown of correlations by age was done, i.e. twin correlations were estimated for pairs aged 18 years or younger versus pairs over 18 years. In MZ twins (N = 383 pairs for twins 18 or younger and 2601 pairs over 18) the correlations were 0.53 (0.46–0.59) and 0.52 (0.49–0.54). In DZ pairs (N = 489 pairs for twins 18 or younger and 2632 pairs over 18) the correlations were 0.26 (0.18–0. 34) and 0.20 (0.17–0.24), consistent with younger pairs showing a somewhat larger resemblance.

The mean IRT neuroticism score was 0.08 (SD = 0.98) with values ranging from − 3.43 to 4.28. There was a small negative correlation of age and neuroticism of − 0.076. There was an effect of sex on neuroticism with women scoring 0.4 standard deviations higher on average than men. In this large sample, both effects are highly significant. The additive genetic, non-additive genetic (dominance) and shared household variance component estimates and their standard errors are given in Table 3, along with the log-likelihood (LL) for each model. Compared to estimates, the standard errors are small, indicating that the additive genetic and dominance components estimates were significant as was the effect of a shared household. Removing either the shared household effect or the dominance effect introduced a worsening of fit.

**Table 3:** Parameter estimates (and SE) for variance components

Model	Log-likelihood	Additive genetic variance	Dominance genetic variance	Shared household variance	Non-genetic (E) variance	Heritability (additive and dominance)	Household^a^
ADE plus household	− 13390.40	0.2449 (0.0145)	0.1822 (0.0175)	0.1210 (0.0100)	0.3656 (0.0135)	0.4271/0.9137 = 0.4674	0.1210/0.9137 = 0.1324
ADE no household	− 13464.28	0.2455 (0.0134)	0.1912 (0.0168)	–	0.4761 (0.0109)	0.4367/0.9128 = 0.4784	–
AE plus household	− 13446.34	0.3454 (0.0111)	–	0.1270 (0.0098)	0.4460 (0.0129)	0.3454/0.9184 = 0.3761	0.1270/0.9184 = 0.1383

Heritability and household columns give the proportion of variance explained by total genetic and household effects

^a^All spouses and their offspring aged 18 years or less

## Discussion

Based on the analysis of neuroticism data in > 30,000 individuals, we conclude that variation in neuroticism is in part explained by genetic influences. Additive and non-additive genetic factors are likely to both contribute to variation and explained respectively 27 and 20% of the total phenotypic variance, i.e. the narrow-sense heritability for neuroticism was 27% and the broad-sense heritability 47%. These estimates were stable under different model specifications, that is, the estimates hardly changed when omitting the significant shared household effect from the model. Genetics of Personality Consortium et al. (2015) estimated in a Dutch subgroup from this cohort with neuroticism and genome-wide single nucleotide polymorphism (SNP) data that 14.7% of the variance in neuroticism was explained by SNPs (CI 0.2–29%). In the UK Biobank data (Smith et al. 2016), where EPQ-Neuroticism was assessed in > 90,000 subjects, a SNP-based heritability estimate for neuroticism of ∼ 15% (SE = 0.7%) was reported and in a publication by Lo et al. (2017) in nearly 60,000 participants from 23 and me, the SNP heritability was 12% (CI 8.7–15%). These estimates account for additive genetic variance and based on our estimate of 27% for narrow-sense heritability, the SNP-based estimates thus capture around 50% of the additive genetic variance in neuroticism.

Often the analysis of pedigree data is limited to estimation of the additive genetic (A) and unique environmental (E) variance components (Docherty et al. 2015). In contrast, twin researchers often explicitly consider common environmental effects, while recognizing that the classical twin design does not allow simultaneous estimation of common environmental (C) and genetic dominance variance (D) components. Keller et al. (2010) observed that increasingly complex extended twin family designs produce more accurate results, and are less sensitive to violations of model assumptions than simpler models. They concluded that researchers interested in characterizing the environment or the composition of genetic variance should use extended designs when possible. Here we used all information on neuroticism from twins and their family members, including distant relatives, and estimated a significant contribution of shared household environment in the presence of genetic dominance. We should note that a shared household effect does not capture the same effects that may be included when modeling common environment in the classical twin design. For example it does not account for any persistent effects of shared environment on resemblance among relatives. We explored alternative definitions of ‘household sharing’ including sharing defined as true when a household was ever shared, household sharing for all siblings including twins and household sharing for twins only. None of these models described the data better than the immediate household definition (shared only when the household is shared at the time of the neuroticism assessment), indicating that persistent shared environment is less likely. Lake et al. (2000) previously analyzed data on neuroticism in extended twin kinships among 45,850 family members from Australia and the United States. Their estimate of broad sense heritability was nearly identical to the estimate obtained in our sample from the Netherlands. However, rather than modeling a household effect of relatives living together, Lake et al. considered the effects of assortative mating and vertical environmental transmission, which will lead to a shared sibling environment. They found that assortative mating was too small to have a substantial effect on the correlation between relatives. In Mendel it is not possible to model and test for the genetic effects of phenotypic assortative mating. We explored if marital cohabitation might explain spousal resemblance and found support for this when comparing younger and older couples. Lake et al. also concluded that there was no evidence for vertical environmental transmission. The estimate of 13% explained variance by shared household in the Dutch data does not primarily arise from vertical transmission and is based on the similarity among spouses, parents and their offspring of < 18 years, as well as siblings and twins under 18 years, who live together in the same household. For a phenotype related to neuroticism, Dolan et al. (2014) obtained support for the effect of one twin’s phenotype on the other twin’s environment in a longitudinal analysis of childhood anxiety. That is, the anxious behavior of one twin contributed to the environment of the co-twin. This process gives rise to genotype-environment covariance, which may produce apparent shared environmental influences, if it is not explicitly modeled. This process is not limited to twins, i.e., the behavior or personality of any family member may contribute to the environment of his or her cohabitants and such covariance between additive genetic and household effects will also contribute to a household effect (Purcell 2002) if it is not modeled. We therefore realize that household effect may in part be attributable to other process other than the simple main effects of ‘true’ shared environment. Within individuals, high neuroticism scores are a risk factor for depression, which is supported by the prediction of major depressive disorder by polygenic neuroticism scores (Genetics of Personality Consortium et al. 2015), and high genetic correlations of neuroticism and depression (Gale et al. 2016; Okbay et al. 2016; Lo et al. 2017). It is possible that not only one’s own neuroticism, but also neuroticism in family members with whom an individual shares a household constitutes a risk factor for depression. To aid in exploring such scenario’s and resolve the interpretation of genetic correlations and causation at multiple explanatory levels, phenotype and genotype data not only from individuals but also from those with whom they share a household or a broader environment are required (e.g., Nivard and Boomsma 2016).

## Appendix

Distribution of age differences between father-offspring and mother-offspring for the age at which the neuroticism survey was completed and distribution of differences in neuroticism scores.
